# Resistance to cyclin-dependent kinase (CDK) 4/6 inhibitors confers cross-resistance to other CDK inhibitors but not to chemotherapeutic agents in breast cancer cells

**DOI:** 10.1007/s12282-020-01150-8

**Published:** 2020-08-28

**Authors:** Ryohei Ogata, Emi Kishino, Wataru Saitoh, Yoshikazu Koike, Junichi Kurebayashi

**Affiliations:** Department of Breast and Thyroid Surgery, Kawasaki Medical School, 577 Matsushima, Kurashiki, Okayama 701-0192 Japan

**Keywords:** Breast cancer, CDK4/6 inhibitor, Resistance, Chemotherapeutic agents, RB

## Abstract

**Background:**

Combined endocrine therapy with a cyclin-dependent kinase (CDK) 4/6 inhibitor has been indicated to improve not only progression-free survival, but also overall survival in patients with hormone receptor (HR)-positive, HER2-negative advanced breast cancer. However, resistance to this combination therapy inevitably develops. How to manage this resistant breast cancer is one of the most important clinical issues. To investigate the mechanisms of action responsible for resistance, we developed breast cancer cells resistant to CDK4/6 inhibitors, and analyzed their biological characteristics and sensitivity to different anticancer agents.

**Methods:**

HR-positive, HER2-negative MCF-7 and KPL-1 breast cancer cells were cultivated in palbociclib (PAL) or abemaciclib (ABE)-added culture medium for over 5 months, and we successfully developed PAL- or ABE-resistant cells. The effects of PAL or ABE on the cell growth, basal RB expression, RB phosphorylation, cell cycle and cell senescence were compared between resistant and parental cells. Effects of the other CDK4/6 inhibitor, different chemotherapeutic agents and estrogen on the cell growth were also examined. The expression levels of cyclin D1, CDK2, CDK4, CDK6, cyclin E1 and estrogen receptor (ER)-ɑ were measured using RT-PCR.

**Results:**

Long-term exposure to up to 200 nM PAL or ABE resulted in the development of PAL- or ABE-resistant MCF-7 or KPL-1 breast cancer cells. Basal expression levels of RB in both resistant cells were down-regulated. Inhibitory effects of either PAL or ABE on RB phosphorylation were reduced in both resistant cells. Accordingly, G1-S cell cycle retardation and cell senescence induced by either inhibitor were also attenuated in both resistant cells. Both resistant cells were cross-resistant to the other CDK4/6 inhibitor but almost as equally sensitive to different chemotherapeutic agents (5-fluorouracil, gemcitabine, paclitaxel, docetaxel, doxorubicin and eribulin) as the parental cells. The mRNA expression level of *CDK6* significantly increased in the resistant MCF-7 cells and that of *Rb1* significantly decreased in the resistant KPL-1 cells. Although both resistant cells were less sensitive to estrogen than the parental cells, the expression levels of ER-ɑ did not significantly change in either.

**Conclusions:**

Our study suggests that acquired resistance to PAL or ABE confers cross-resistance to the other CDK4/6 inhibitor but not to chemotherapeutic agents in HR-positive, HER2-negative breast cancer cells. Down-regulation of basal RB expression and normalized RB phosphorylation reduced by CDK4/6 inhibitors may be responsible for the attenuated anti-cell growth effects of the inhibitors.

**Electronic supplementary material:**

The online version of this article (10.1007/s12282-020-01150-8) contains supplementary material, which is available to authorized users.

## Introduction

Hormone receptor (HR)-positive, human epidermal growth factor 2 (HER2)-negative breast cancer is the most common subtype of breast cancer. Recently, combined endocrine therapy with cyclin-dependent kinase (CDK) 4/6 inhibitors has been used as the first- or second-line treatment for patients with HR-positive, HER2-negative metastatic breast cancer. These treatments are suggested to improve not only progression-free survival, but also overall survival compared with endocrine therapy alone. However, some HR-positive, HER2-positive breast cancers are de novo resistant to the combined treatments. Furthermore, acquired resistance to the combined treatments frequently develops. Therefore, the optimal treatment strategies for breast cancer resistant to the combined therapies remain one of most important unanswered questions in the management of breast cancer [1].

The mechanisms of action responsible for de novo or acquired resistance to CDK4/6 inhibitors have been investigated in preclinical and translational studies in recent years. They revealed that there are at least two mechanisms. One is dysregulation of cell cycle machineries such as the loss or dysfunction of retinoblastoma protein (RB), CDK6 amplification and upregulation of the cyclin E/CDK2 pathway [2]. The other is a cell cycle-independent mechanism such as upregulation of the PI3K/AKT/mTOR or fibroblast growth factor receptor (FGFR) pathway [2]. Indeed, several recent translational studies demonstrated that some breast cancers acquired these alterations during CDK4/6 inhibitor therapy [3–5].

To elucidate the mechanisms of action responsible for CDK4/6 inhibitor resistance and to explore optimal treatment strategies against such resistant breast cancers, we conducted this preclinical study. First, we developed in vitro models of acquired resistance to two different CDK4/6 inhibitors, palbociclib (PAL) and abemaciclib (ABE), using two different HR-positive HER2-negative breast cancer cell lines. Second, the anti-cell growth activity of different anti-tumor agents, such as the other CDK4/6 inhibitor and cytotoxic chemotherapeutic agents, was assessed. Third, to explore biological alterations in the resistant breast cancer cells, estrogen responsiveness and changes in the expression levels of cell cycle-related factors and estrogen receptor (ER)-ɑ were examined.

## Materials and methods

### Reagents

PAL and ABE were obtained from AdooQ BIOSCIENCE (Irvine, CA, USA) and LKT Laboratories, Inc. (St. Paul, MN, USA), respectively. 17β-estradiol (E2) was obtained from Sigma-Aldrich (St. Louis, MO, USA). All cytotoxic chemotherapeutic agents (5-fluorouracil [5-FU], gemcitabine [GEM], doxorubicin [DOX], paclitaxel [PAC], docetaxel [DOC] and eribulin [ERI]) were obtained from Sigma-Aldrich.

### Breast cancer cell lines and cell culture

MCF-7 cells were kindly provided by the late Robert B. Dickson, Lombardi Cancer Research Center, Georgetown University, Washington DC, USA. KPL-1 cells were established in our laboratory [6]. MCF-7 cells express both ER-ɑ and progesterone receptor (PR). KPL-1 cells express ER-ɑ but not PR. MCF-7 cells are more sensitive to estrogen than KPL-1 cells. KPL-1 cells can grow under estrogen-deprived medium, but MCF-7 cells cannot. Both cell lines were cultivated in D-MEM supplemented with 10% fetal bone serum (FBS).

### Development of breast cancer cells resistant to CDK4/6 inhibitors

Either MCF-7 or KPL-1 cells were cultivated under RPMI1640 medium supplemented with 5% FBS and indicated concentrations of PAL or ABE for 5 months. Concentrations of PAL or ABE were increased step-by-step as follows: 100 nM during the first month, 125 nM during the second month, 150 nM during the third month, 175 nM during the fourth month and 200 nM during the fifth month. Both MCF-7 and KPL-1 cells steadily grew in the PAL- or ABE-added medium. MCF-7 cells growing in 200 nM PAL- and ABE-added medium were denoted as MR-P and MR-A cells, respectively. KPL-1 cells growing in 200 nM PAL- or ABE-added medium were denoted as KR-P and KR-A cells, respectively. MCF-7 and KPL-1 cells growing in the standard medium were denoted as MS and KS cells, respectively.

### Cell growth assay

To examine cell growth, breast cancer cells were seeded on 24-well plates (SB Medical, Tokyo, Japan) and grown in RPMI1640 medium supplemented with 5% FBS at 37 °C in a 5% CO_2_ atmosphere for 1 day. After washing with phosphate-buffered saline (PBS, Nissui Co., Tokyo, Japan), the cells were cultured in estrogen-deprived medium consisting of phenol red-free RPMI1640 (Life Technologies, Carlsbad, CA, USA) supplemented with 5% dextran-coated charcoal-treated FBS (GE Healthcare HyClone, Tokyo, Japan) plus 1 nM E2 and the indicated concentrations of PAL, ABE and chemotherapeutic agents for 3 days. For the E2-sensitivity assay, the cells were cultured in the estrogen-deprived medium plus the indicated concentrations of E2 for 3 days. Then, the cells were harvested and counted using a Coulter counter (Coulter Electronics, Harpenden, UK) [7].

### Cell cycle and cell senescence assays

To investigate cell cycle progression, harvested cells were stained with propidium iodide using the CycleTest Plus DNA Reagent kit (Becton–Dickinson, San Jose, CA, USA) according to the manufacturer’s recommendations. Flow cytometry was performed using a FACSCalibur flow cytometer (Becton–Dickinson) and the DNA histogram was analyzed using CELLQuest version 6.0 (Becton–Dickinson) [7].

Senescence was measured by the SA-β gal staining kit (Millipore, Billerica, MA, USA) according to the manufacturer’s protocol. In brief, cells were plated at a low density of 2,000 cells in each well of 12-well plates, and treated with the indicated concentrations of PAL or ABE for 3 days. Cells were then washed with PBS, fixed and stained with the SA-β gal solution (Millipore) for 4 hours or overnight. Senescent cells were quantified by counting 100 cells in 3 different fields for each replicate [7].

### Western blot analysis

Cells were lysed for protein extraction using Pierce RIPA Buffer with protease inhibitor and phosphatase inhibitor (Thermo Fisher Scientific, Waltham, MA, USA). The total protein concentration was measured using the Pierce BCA Protein Assay kit (Thermo Fisher Scientific). Isolated proteins were separated by 5–20% SDS-PAGE and transferred to an Amersham Hybond PVDF (GE Healthcare UK, Buckinghamshire, UK). Membranes were blocked with blocking buffer at room temperature for 1 hour and then subjected to immunoblotting using primary antibodies at 4 °C overnight, followed by incubation with secondary antibodies at room temperature for 1 hour. Labeled protein was visualized using the ECL Prime Western Blotting Detection Reagent (GE Healthcare Japan, Tokyo, Japan), with the expression of β-actin as the internal standard. The expression levels were measured using Quantity One 1-D software ver.4.5 (BIORAD, Tokyo, Japan) [7].

Rabbit antibodies against RB (mAb #9313) and phosphorylated RB (mAb #8516) were purchased from Cell Signaling Technologies (Danvers, MA, USA). Mouse polyclonal antibody against β-actin was from Sigma Aldrich. Secondary antibodies, goat anti-rabbit lgG-HRP and goat anti-mouse lgG-HRP, were purchased from Santa Cruz Biotechnology (Dallas, Texas, USA) [7].

### RNA isolation and quantitative reverse-transcription polymerase chain reaction (RT-PCR)

Total RNA from the cells was extracted using an RNeasy MiniKit (QIAGEN GmbH, Hilden, Germany) according to the manufacturer’s instructions and cDNA synthesis was performed using a ReverTra Ace qPCR RT kit (TOYOBO, Tokyo, Japan). Quantitative real-time PCR analysis of *Rb1*, *CDK2, CDK4, CDK6, cyclin D 1 (CCND1), cyclin E 1 (CCNE1) and ER-ɑ* mRNA was performed on cDNA using TaqMan gene expression assays according to the manufacturer’s instructions (Applied Biosystems, Life Technologies, Waltham, MA, USA) and a 7500 Real-Time PCR System (Applied Biosystems). Each amplification reaction was performed in duplicate, and the average of the two threshold cycles was used to calculate the amount of transcripts in the sample. The mRNA quantification was expressed, in arbitrary units, as the ratio of the sample quantity to the calibrator or to the mean values of the control samples. All values were normalized to an endogenous control, *ACTB.* A change in the amount of transcript to greater than 2 or less than 0.5 was considered to be significant [7].

### Statistical analysis

All values are expressed as the mean ± SE. Analysis of variance analyzed by the Fisher’s protected least significant difference (PLSD) test with StatView computer software (ATMS Co., Tokyo, Japan) was used to compare differences between two groups. A two-sided P value less than 0.05 was considered significant.

## Results

### Establishment of PAL- or ABE-resistant breast cancer cells

**Fig. 1:**
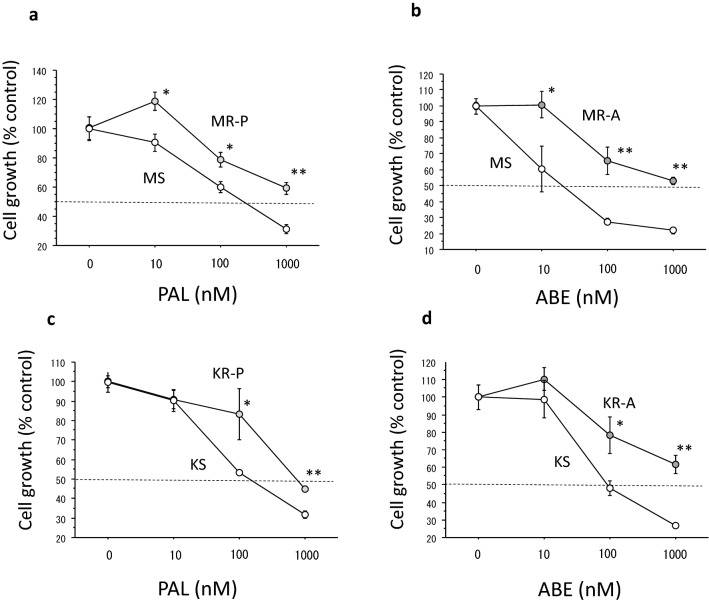
Growth inhibitory curves of PAL or ABE in MCF-7 cells or KPL-1 cells. **a** Those of PAL in MS cells and MR-P cells. **b** Those of ABE in MS cells and MR-A cells. **c** Those of PAL in KS cells and KR-P cells. **d** Those of ABE in KS cells and KR-A cells. The values are the mean ± SE. Open circles, sensitive cells; and grey circles, resistant cells. *P < 0.05; **P < 0.01 for comparison between the sensitive and resistant cells at each concentration

As shown in Table 1, the 50% growth-inhibitory concentrations [IC_50_s] in MR-P cells for PAL and in MR-A cells for ABE were approximately 9 and 16 times higher than those in MS cells, respectively. The IC_50_s in KR-P cells for PAL and in KR-A cells for ABE were approximately 3 and 28 times higher than those in KS cells, respectively. Growth inhibitory curves of the respective resistant and sensitive cells are shown in Fig. 1.

**Table 1:** IC_50_s of PAL and ABE in breast cancer cells (mean ± SE)

	PAL (nM)	ABE (nM)
MCF-7 cells		
MS cells	251 ± 44	94 ± 5
MR-P cells	2194 ± 589*	600 ± 200*
MR-A cells	850 ± 149*	1507 ± 288**
KPL-1 cells		
KS cells	202 ± 33	220 ± 40
KR-P cells	610 ± 74**	1055 ± 292*
KR-A cells	1025 ± 166**	6158 ± 538**

*P < 0.05, **P < 0.01

### Cross-resistance to the other CDK4/6 inhibitor

**Fig. 2:**
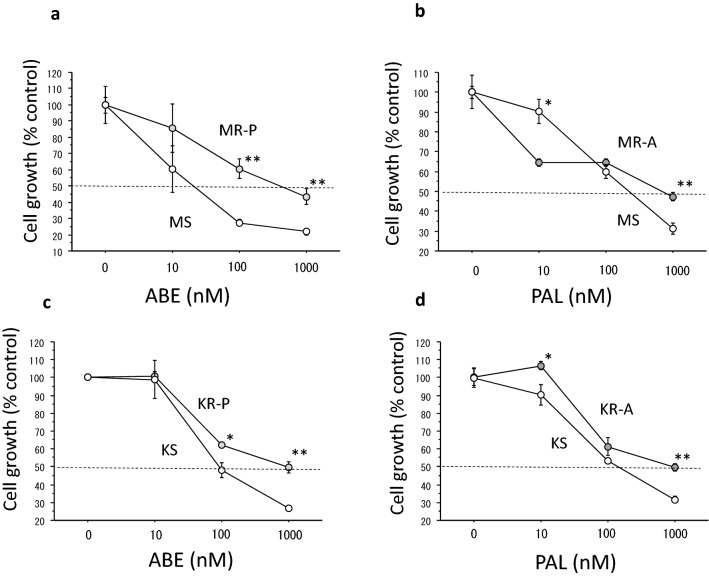
Cross-resistance between PAL and ABE. Growth inhibitory curves of PAL or ABE in MCF-7 cells or KPL-1 cells. **a** Those of ABE in MS cells and MR-P cells. **b** Those of PAL in MS cells and MR-A cells. **c** Those of ABE in KS cells and KR-P cells. **d** Those of PAL in KS cells and KR-A cells. The values are the mean ± SE. Open circles, sensitive cells; and grey circles, resistant cells. *P < 0.05; **P < 0.01 for comparison between the sensitive and resistant cells at each concentration

As shown in Table 1, the IC_50_s in MR-P and KR-P for ABE were approximately 6 and 5 times higher than those in MS and KS cells, respectively. The IC_50_s in MR-A and KR-A cells for PAL were approximately 3 and 5 times higher than those in MS and KS cells, respectively. Growth inhibitory curves of the respective resistant and sensitive cells are shown in Fig. 2.

### Anti-tumor activity of chemotherapeutic agents against the resistant and sensitive cells

**Fig. 3:**
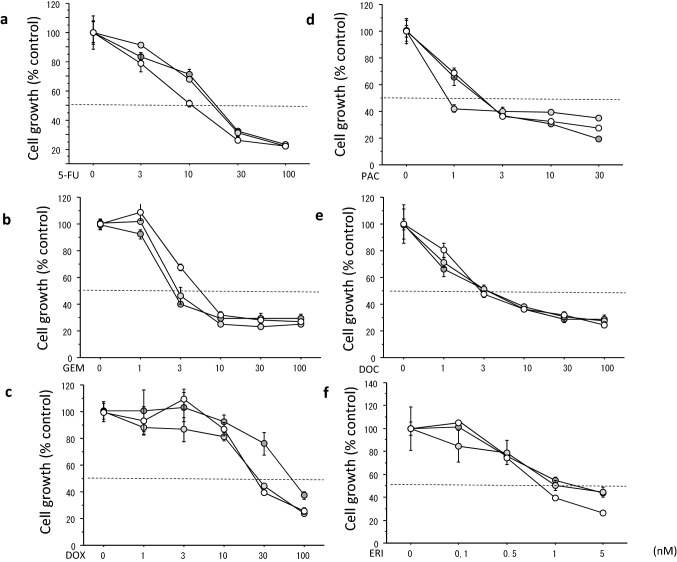
Growth inhibitory curves of chemotherapeutic agents in MCF-7 cells sensitive or resistant to PAL or ABE. Cells were treated for 3 days with 5-FU (**a**), GEM (**b**), DOX (**c**), PAC (**d**), DOC (**e**) and ERI (**f**). The values are the mean ± SE. Open circles, MS cells; light grey circles, MR-P cells; and dark grey circles, MR-A cells

**Fig. 4:**
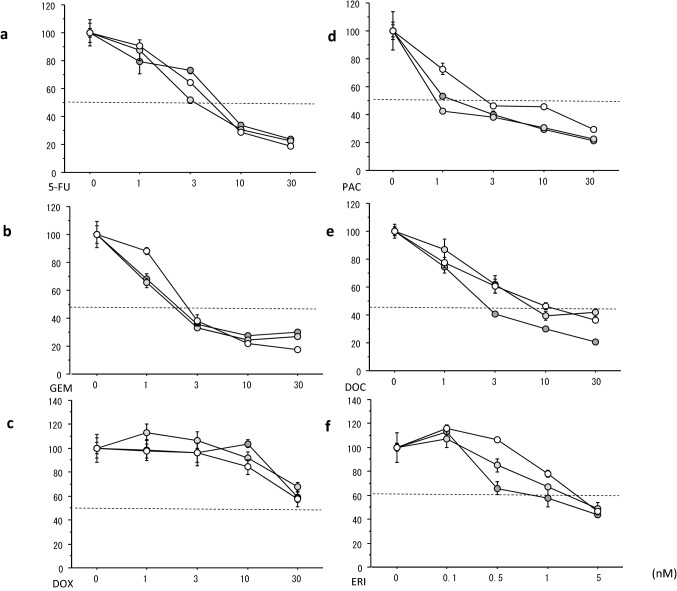
Growth inhibitory curves of chemotherapeutic agents in KPL-1 cells sensitive or resistant to PAL or ABE. Cells were treated for 3 days with 5-FU (**a**), GEM (**b**), DOX (**c**), PAC (**d**), DOC (**e**) and ERI (**f**). The values are the mean ± SE. Open circles, KS cells; light grey circles, KR-P cells; and dark grey circles, KR-A cells

As shown in Table 2, the IC_50_s of six chemotherapeutic agents among MR-P, MR-A and MS cells were almost identical. There was no significant difference among them. Similarly, the IC_50_s of six different agents among KR-P, KR-A and KS cells were almost identical. Growth inhibitory curves of the respective resistant and sensitive cells are shown in Figs. 3, 4.

**Table 2:** IC50s of chemotherapeutic agents in breast cancer cells (mean ± SE)

MCF-7 cells			
Agents (nM)	MS cells	MR-P cells	MR-A cells
5-FU	7.3 ± 0.7	8.0 ± 0.2	6.4 ± 0.4
GEM	8.6 ± 1.2	8.2 ± 0.6	9.4 ± 0.7
DOX	36.4 ± 5.6	36.2 ± 2.9	32.8 ± 3.7
PAC	3.7 ± 0.4	1.3 ± 0.6	3.7 ± 1.0
DOC	6.6 ± 0.9	6.6 ± 0.2	5.2 ± 0.1
ERI	2.7 ± 0.2	1.9 ± 0.7	2.2 ± 0.1

KPL-1 cells			
Agents (nM)	KS cells	KR-P cells	KR-A cells
5-FU	6.9 ± 0.2	7.1 ± 1.3	6.1 ± 1.8
GEM	5.5 ± 0.3	3.4 ± 1.1	5.6 ± 0.9
DOX	20.2 ± 2.6	31.9 ± 4.1	25.9 ± 4.1
PAC	6.0 ± 1.3	3.2 ± 1.3	3.2 ± 0.7
DOC	10.8 ± 1.6	15.9 ± 3.6	8.8 ± 0.70
ERI	7.5 ± 0.2	8.1 ± 0.9	7.0 ± 0.9

### Effects of PAL and ABE on cell cycle progression and cell senescence in the resistant and sensitive cells

As it has been well documented that the anti-cell growth activity of CDK4/6 inhibitors depends on the G1-S blockade and cell senescence [8], the effects of PAL and ABE were investigated in the PAL- or ABE-resistant cells. Both PAL and ABE induced less G1-S retardation in MR-P or KR-P cells than in MS or KS cells (Online Resource 1). Similarly, both PAL and ABE induced less cell senescence in MR-P or KR-P cells than in MS or KS cells (Online Resource 1).

### Expression levels of basal and phosphorylated RB in the resistant and sensitive cells

**Fig. 5:**
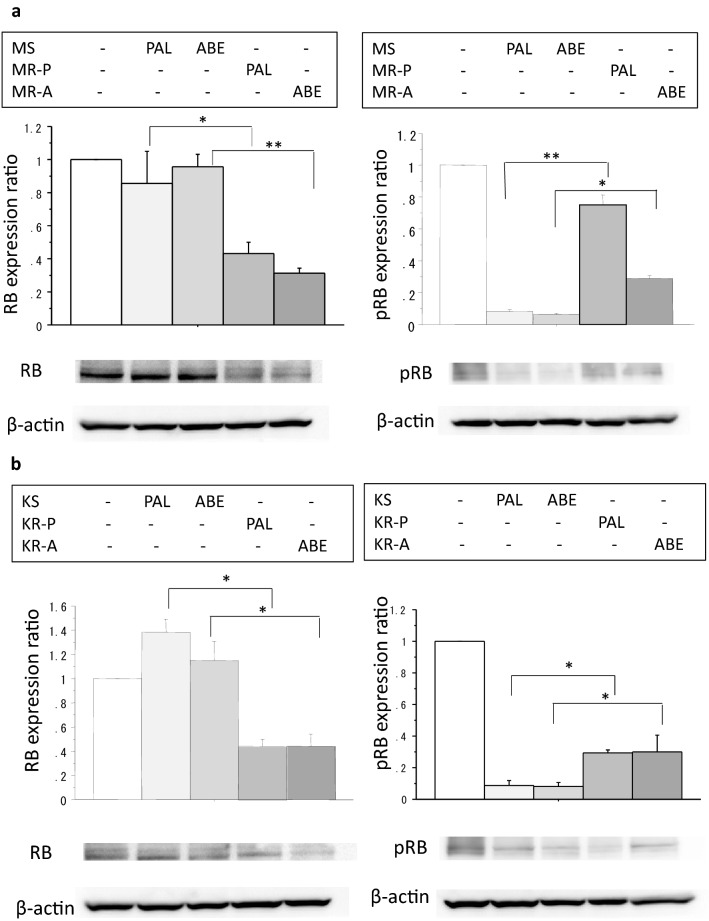
The protein expression levels of RB (**a**) and phosphorylated RB (**b**) altered by PAL or ABE in MS cells as the control (white bars), PAL-treated MS cells (the lightest grey bars), ABE-treated MS cells (the second lightest bars), PAL-treated MR-P cells (the third lightest bars) and ABE-treated MR-A cells (the darkest bars). The protein expression levels of RB (**c**) and phosphorylated RB (**d**) altered by PAL or ABE in KS cells as the control (white bars), PAL-treated KS cells (the lightest grey bars), ABE-treated KS cells (the second lightest bars), PAL-treated KR-P cells (the third lightest bars) and ABE-treated KR-A cells (the darkest bars). Expression levels were measured by Western blotting as described in Materials and Methods. Representative blots are shown. Values were analyzed after normalization to the controls and expressed as the mean ± SE. The expression level of each molecule in control cells was defined as 1. *P < 0.05; **P < 0.01

As the expression levels of basal and phosphorylated RB regulate the cyclin D-CDK4/6-Rb-E2F pathway [8], their protein expression was measured by Western blotting using the resistant and sensitive cells. Basal expression levels of RB were significantly down-regulated in MR-P and MR-A cells compared with those in MS cells (Fig. 5a). Similarly, they were significantly down-regulated in KR-P and KR-A cells compared with those in KS cells (Fig. 5b). Inhibitory effects of PAL or ABE on RB phosphorylation were partially restored in MR-P and MR-A cells compared with MS cells (Fig. 5a). Similarly, they were partially restored in KR-P and KR-A cells compared with KS cells (Fig. 5b).

### Changes in the expression levels of cyclins and CDKs in the resistant cells

As the basal expression levels of *CCND1*, *CCNE1*, *CDK2*, *CDK4* and *CDK6* may influence RB phosphorylation [8], their mRNA expression levels were measured by RT-PCR in the resistant and sensitive cells. The mRNA expression level of *CDK6* was significantly up-regulated in MR-P and MR-A cells compared with that in MS cells (Online Resource 2). That of *Rb1* was significantly down-regulated in KR-P and KR-A cells compared with that in KS cells (Online Resource 2). Those of the other molecules were not significantly changed in the resistant cells (Online Resource 2).

### Sensitivity to E2 and ER-α expression in the resistant and sensitive cells

Resistance to CDK4/6 inhibitors was suggested to affect estrogen sensitivity [9, 10]; therefore, the growth-promoting effects of E2 on the resistant and sensitive cells were investigated. These effects were significantly down-regulated in MR-P, MR-A, KR-P and KR-A cells compared with in MS and K-P cells (Online Resource 3). However, the mRNA expression levels of ER-α were similar between resistant and sensitive cells (Online Resource 2).

## Discussion

We successfully developed two different HR-positive, HER2-negative cell lines, MCF-7 and KPL-1, resistant to two different CDK4/6 inhibitors, PAL and ABE, using long-time exposure to PAL or ABE by increasing their concentration in a stepwise manner. MCF-7 cells are well known to be highly sensitive to estrogen. Their growth depends on estrogen-supplementation both in vitro and in vivo. In contrast, our home-made KPL-1 cells are relatively sensitive to estrogen, but their growth does not depend on estrogen-supplementation both in vitro and in vivo. The KPL-1 cells originated from a patient with recurrent breast cancer clinically resistant to tamoxifen and medroxyprogesterone acetate [6]. These findings suggest that MCF-7 and KPL-1 cell lines were different in terms of estrogen responsiveness.

Parental MCF-7 and KPL-1 cells are sensitive to both PAL and ABE in terms of cell growth inhibition. Parental MCF-7 cells were slightly more sensitive to ABE than parental KPL-1 cells (Table 1). Based on the IC_50_s of PAL or ABE, the PAL- or ABE-resistant MCF-7 or KPL-1 cells were 3 to 16 times more resistant to PAL and ABE, respectively (Table 1). Of note, cross-resistance between PAL and ABE was demonstrated in both MCF-7 and KPL-1 cells (Table 1). Based on the IC_50_s of PAL or ABE, the PAL- or ABE-resistant MCF-7 or KPL-1 cells were 3 to 6 times more cross-resistant to APL and ABE, respectively (Table 1). Cross-resistance among CDK4/6 inhibitors has been reported in both preclinical and clinical conditions [1, 10–12].

As previously mentioned, how to manage breast cancers resistant to CDK4/6 inhibitors is one of the most important clinical issues. Based on preclinical studies suggesting that up-regulation of the PI3K-AKT-mTOR signaling pathway causes acquired resistance to CDK4/6 inhibitors, the mTOR inhibitor everolimus and PI3K inhibitor alpelisib have been clinically used to manage CDK4/6 inhibitor-resistant breast cancer [1, 13]. However, their anti-tumor activity remains to be clarified. In addition, based on preclinical studies suggesting that activation mutations or amplification of the FGFR pathway cause acquired resistance to CDK4/6 inhibitors, the FGFR tyrosine kinase inhibitor lucitanib has been clinically tested in patients with advanced breast cancer [14].

Physicians frequently prescribe cytotoxic chemotherapeutic agents to patients with advanced breast cancer resistant to CDK4/6 inhibitors. It can be hypothesized that breast cancer resistant to CDK4/6 inhibitors acquires cross-resistance to chemotherapeutic agents and reduces post-progression survival after CDK4/6 inhibitor therapies. However, recent analyses revealed that treatments with CDK4/6 inhibitors and endocrine therapy provided progression-free survival benefits in addition to overall survival benefits in patients with advanced breast cancer compared with endocrine therapy alone [1]. This suggests that breast cancer resistant to CDK4/6 inhibitors retains sensitivity to chemotherapeutic agents. To clarify this, we compared antitumor activity of six chemotherapeutic agents commonly used in clinics among breast cancer cells sensitive or resistant to CDK4/6 inhibitors in this study. As expected, the anti-tumor activity of all chemotherapeutic agents did not change in the two breast cancer cells lines resistant to PAL or ABE compared with their respective parental cell lines (Table 2). Similar findings were recently reported, but only two chemotherapeutic agents were assessed [10].

The mechanisms of action responsible for acquired resistance to CDK4/6 inhibitors have been extensively investigated in recent years. One possible mechanism is the alteration of cell cycle machineries. To clarify this hypothesis, we investigated basal expression levels of cell cycle-related molecules in breast cancer cells sensitive or resistant to PAL or ABE in this study. The basal RB expression was significantly down-regulated in both resistant cell lines compared with that in parental cells (Fig. 4). Moreover, the reduced expression levels of phosphorylated RB were restored in the resistant cells compared with those in parental cells (Fig. 4). It is well known that the dysfunction or loss of RB correlates with resistance to CDK4/6 inhibitors in preclinical and clinical studies [2, 15]. RB phosphorylation also plays an essential role in the cyclin D-CDK4/6-RB-E2F cell cycle machineries. These findings strongly suggest that reduced basal RB expression and normalized RB phosphorylation make breast cancer cells resistant to CDK4/6 inhibitors.

On the other hand, the basal mRNA expression level of *CDK6* was up-regulated in the resistant MCF-7 cells but not in the resistant KPL-1 cells (Online Resource 2). CDK6 amplification was reported to be correlated with resistance to CDK4/6 inhibitors in preclinical studies [9]. This suggests that altered cell cycle machineries, such as the increased expression of CDK6, plays a role in the acquisition of resistance to CDK4/6 inhibitors in some breast cancers.

Recent preclinical studies suggested that the acquisition of resistance to CDK4/6 inhibitors renders cells insensitive to estrogen because of reduced ER-ɑ expression [9, 10]. To clarify this hypothesis, we investigated estrogen sensitivity in the CDK4/6 inhibitor-sensitive or -resistant breast cancer cells in this study. Growth-promoting effects of E2 were significantly reduced in the resistant cells compared with those in the sensitive cells in both models (Online Resource 3). This phenomenon was slightly more marked in KPL-1 cells than in MCF-7 cells. This may be explained by the basal estrogen sensitivity being lower in KPL-1 cells than in MCF-7 cells, as previously described [6]. To elucidate the mechanisms of action responsible for the estrogen insensitivity in the resistant cells, ER-ɑ expression was compared between the resistant and sensitive cells. However, expression was unchanged in both the MCF-7 and KPL-1 models. These findings support the hypothesis that acquired resistance to CDK4/6 inhibitors reduces both estrogen sensitivity and sensitivity to endocrine therapy, which weakens the antitumor activity of combined CDK4/6 inhibitor and endocrine therapy. Further preclinical and clinical studies are needed to clarify this hypothesis.

There are some limitations in this experimental study. All experiments were performed in vitro and only two breast cancer cell lines were used. However, in this study using two different HR-positive, HER2-negative cell lines resistant to PAL or ABE, the resistant cells developed cross-resistance to the other CDK4/6 inhibitor but not to several chemotherapeutic agents commonly used for the treatment of advanced breast cancer resistant to CDK4/6 inhibitors. Reduced basal expression levels of RB and normalized expression levels of phosphorylated RB may explain the reduced antitumor activity associated with the decrease in G1-S blockade and cell senescence in the resistant cells.

## Electronic supplementary material

Below is the link to the electronic supplementary material.

Supplementary file1 (PDF 458 kb)

Supplementary file2 (PDF 504 kb)

Supplementary file3 (PDF 444 kb)

## References

[1] Spring LM, Wander SA, Andre F, Moy B, Turner NC, Bardia A (2020). Cyclin-dependent kinase 4 and 6 inhibitors for hormone receptor-positive breast cancer: past, present, and future. Lancet.

[2] Pandey K, An HJ, Kim SK, Lee SA, Kim S, Lim SM (2019). Molecular mechanisms of resistance to CDK4/6 inhibitors in breast cancer: a review. Int J Cancer.

[3] O'Leary B, Cutts RJ, Liu Y, Hrebien S, Huang X, Fenwick K (2018). The genetic landscape and clonal evolution of breast cancer resistance to palbociclib plus fulvestrant in the PALOMA-3 trial. Cancer Discov.

[4] Turner NC, Liu Y, Zhu Z, Loi S, Colleoni M, Loibl S (2019). Cyclin E1 expression and palbociclib efficacy in previously treated hormone receptor-positive metastatic breast cancer. J Clin Oncol.

[5] Formisano L, Lu Y, Servetto A, Hanker AB, Jansen VM, Bauer JA (2019). Aberrant FGFR signaling mediates resistance to CDK4/6 inhibitors in ER+ breast cancer. Nat Commun.

[6] Kurebayashi J, Kurosumi M, Sonoo H (1995). A new human breast cancer cell line, KPL-1 secretes tumour-associated antigens and grows rapidly in female athymic nude mice. Br J Cancer.

[7] Kishino E, Ogata R, Saitoh W, Koike Y, Ohta Y, Kanomata N (2019). Anti-cell growth and anti-cancer stem cell activity of the CDK4/6 inhibitor palbociclib in breast cancer cells. Breast Cancer.

[8] Klein ME, Kovatcheva M, Davis LE, Tap WD, Koff A (2018). CDK4/6 Inhibitors: the mechanism of action may not be as simple as once thought. Cancer Cell.

[9] Yang C, Li Z, Bhatt T, Dickler M, Giri D, Scaltriti M (2017). Acquired CDK6 amplification promotes breast cancer resistance to CDK4/6 inhibitors and loss of ER signaling and dependence. Oncogene.

[10] Iida M, Toyosawa D, Nakamura M, Tsuboi K, Tokuda E, Niwa TI (2020). Decreased ER dependency after acquired resistance to CDK4/6 inhibitors. Breast Cancer.

[11] Iida M, Nakamura M, Tokuda E, Toyosawa D, Niwa T, Ohuchi N (2019). The p21 levels have the potential to be a monitoring marker for ribociclib in breast cancer. Oncotarget.

[12] de Leeuw R, McNair C, Schiewer MJ, Neupane NP, Brand LJ, Augello MA (2018). MAPK reliance via acquired CDK4/6 inhibitor resistance in cancer. Clin Cancer Res.

[13] André F, Ciruelos E, Rubovszky G, Campone M, Loibl S, Rugo HS (2019). Alpelisib for *PIK3CA*-mutated, hormone receptor-positive advanced breast cancer. N Engl J Med.

[14] Hui R, Pearson A, Cortes J, Campbell C, Poirot C, Azim HA (2020). Lucitanib for the treatment of HR^+^/HER2^-^ metastatic breast cancer: results from the multicohort phase II FINESSE study. Clin Cancer Res.

[15] Vijayaraghavan S, Karakas C, Doostan I, Chen X, Bui T, Yi M (2017). CDK4/6 and autophagy inhibitors synergistically induce senescence in Rb positive cytoplasmic cyclin E negative cancers. Nat Commun.

