# Supplementary material for: Resistance to cyclin-dependent kinase (CDK) 4/6 inhibitors confers cross-resistance to other CDK inhibitors but not to chemotherapeutic agents in breast cancer cells
Source: Breast Cancer. 2020 Aug 28;28(1):206–15. doi: 10.1007/s12282-020-01150-8 (PMC7796879; doi:10.1007/s12282-020-01150-8)
Supplement: Supplementary file 1 — Supplementary file1 (PDF 458 kb) [file 12282_2020_1150_MOESM1_ESM.pdf]

Online resource 1-1

Resistance to cyclin-dependent kinase (CDK) 4/6 inhibitors confers cross-resistance to other CDK inhibitors but not to chemotherapeutic agents in breast cancer cells, Breast Cancer, Ogata R, et al., Kawasaki medical School, kure@med.Kawasaki-m.ac.jp

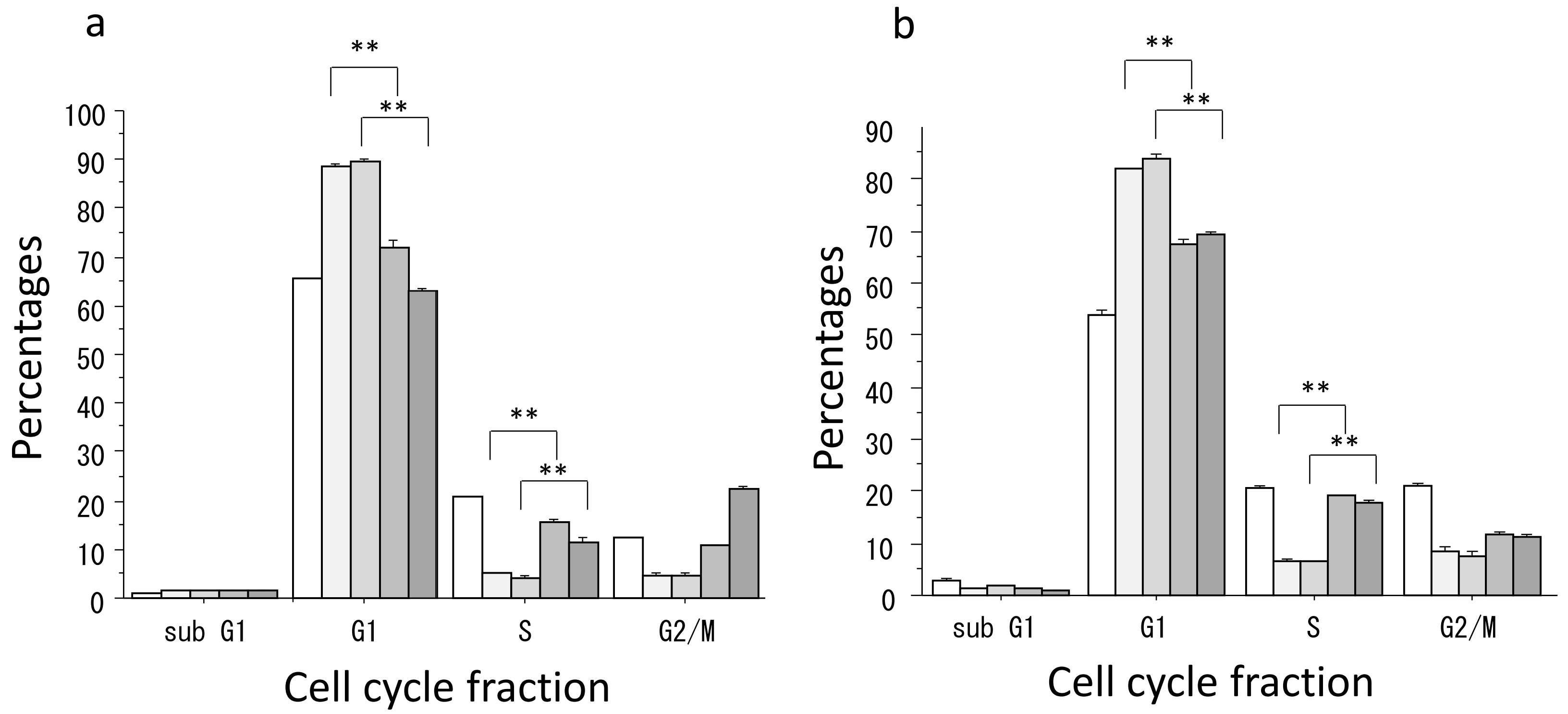

Online Resource 1-2

Resistance to cyclin-dependent kinase (CDK) 4/6 inhibitors confers cross-resistance to other CDK inhibitors but not to chemotherapeutic agents in breast cancer cells, Breast Cancer, Ogata R, et al., Kawasaki medical School, kure@med.Kawasaki-m.ac.jp

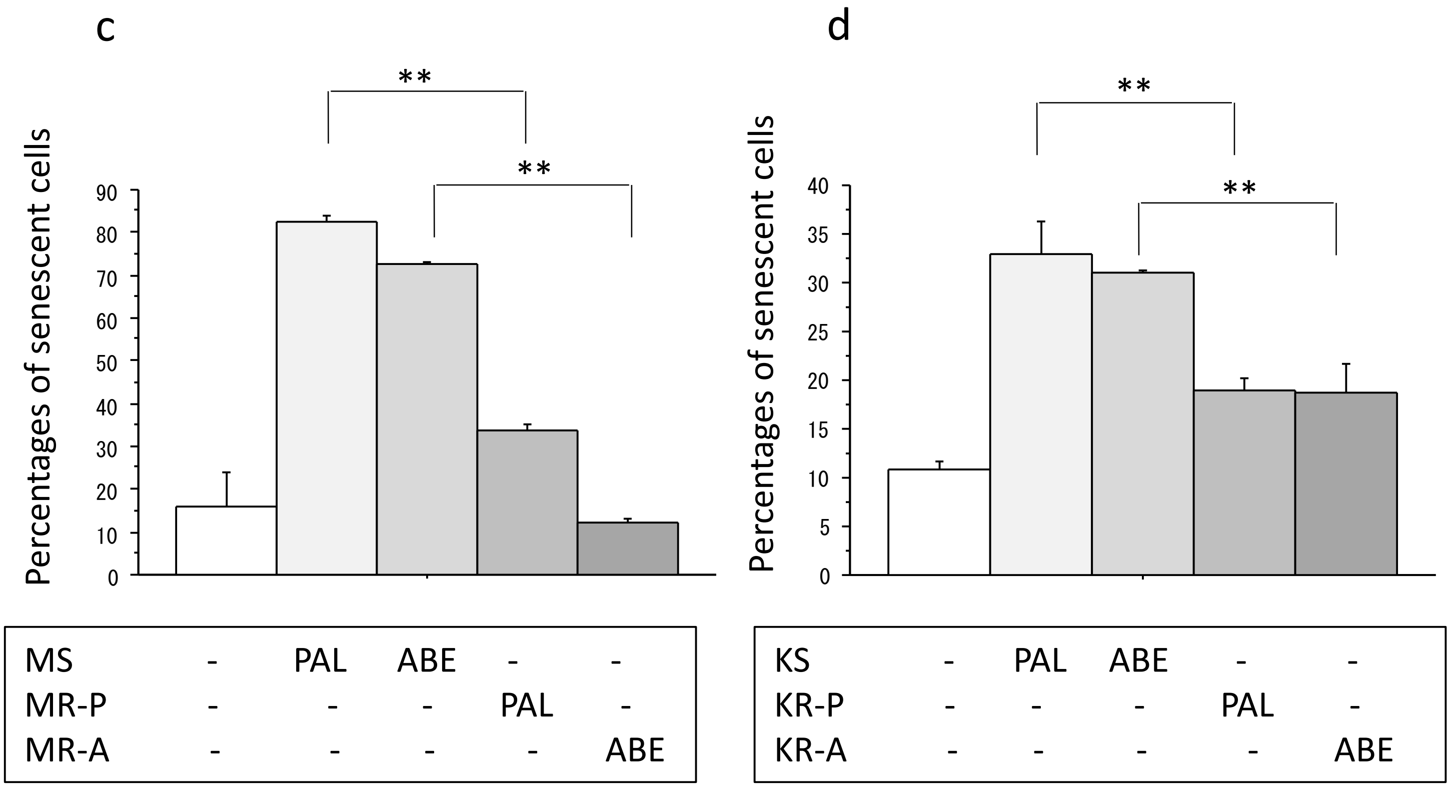

Online Resource 1

a. Effects of PAL or ABE on cell cycle progression in MS cells as the control (white bars), PAL-treated MS cells (the lightest grey bars), ABE-treated MS cells (the second lightest bars), PAL-treated MR-P cells (the third lightest bars) and ABE-treated MR-A cells (the darkest bars).

b. Those in KS cells as the control (white bars), PAL-treated KS cells (the lightest grey bars), ABE-treated KS cells (the second lightest bars), PAL-treated KR-P cells (the third lightest bars) and ABE-treated KR-A cells (the darkest bars).

Cells were treated with 100 nM PAL or ABE for two days. Percentages of cells at each cell cycle phase were analyzed as described in Materials and Methods. Increases in the G1 fraction and decreases in the S fraction, that is a G1-S blockade, induced by PAL or ABE were diminished in all resistant cells.

c. Effects of PAL or ABE on cell senescence in MS cells as the control (white bars), PAL-treated MS cells (the lightest grey bars), ABE-treated MS cells (the second lightest bars), PAL-treated MR-P cells (the third lightest bars) and ABE-treated MR-A cells (the darkest bars).

d. Those in KS cells as the control (white bars), PAL-treated KS cells (the lightest grey bars), ABE-treated KS cells (the second lightest bars), PAL-treated KR-P cells (the third lightest bars) and ABE-treated KR-A cells (the darkest bars).

Cells were treated with 100 nM PAL or ABE for three days. The percentages of β-galactosidase-positive cells were defined as senescent cells, and analyzed as described in Materials and Methods. The values are the mean ± SE. * $P < 0.05$; ** $P < 0.01$.
