# Supplementary material for: Resistance to cyclin-dependent kinase (CDK) 4/6 inhibitors confers cross-resistance to other CDK inhibitors but not to chemotherapeutic agents in breast cancer cells
Source: Breast Cancer. 2020 Aug 28;28(1):206–15. doi: 10.1007/s12282-020-01150-8 (PMC7796879; doi:10.1007/s12282-020-01150-8)
Supplement: Supplementary file 2 — Supplementary file2 (PDF 504 kb) [file 12282_2020_1150_MOESM2_ESM.pdf]

Online Resource 2-1

Resistance to cyclin-dependent kinase (CDK) 4/6 inhibitors confers cross-resistance to other CDK inhibitors but not to chemotherapeutic agents in breast cancer cells, Breast Cancer, Ogata R, et al., Kawasaki medical School, kure@med.Kawasaki-m.ac.jp

a

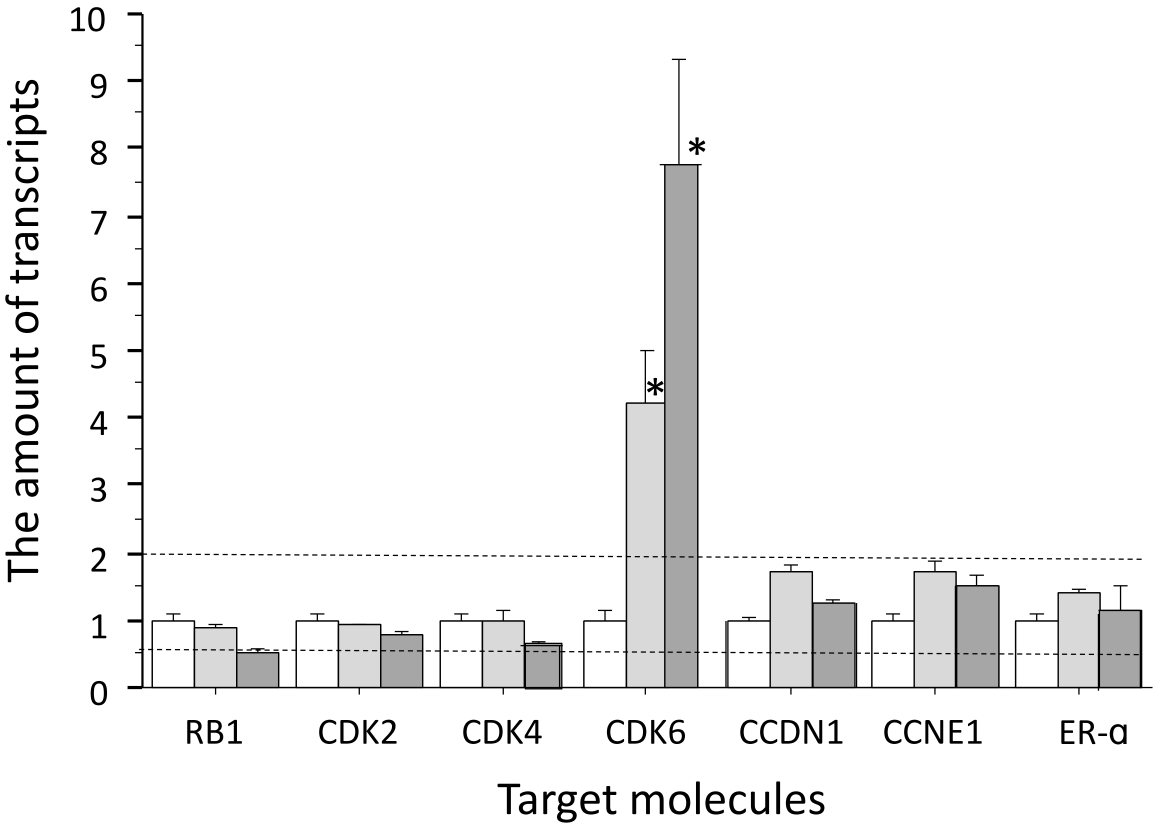

Online Resource 2-2

Resistance to cyclin-dependent kinase (CDK) 4/6 inhibitors confers cross-resistance to other CDK inhibitors but not to chemotherapeutic agents in breast cancer cells, Breast Cancer, Ogata R, et al., Kawasaki medical School, kure@med.Kawasaki-m.ac.jp

b

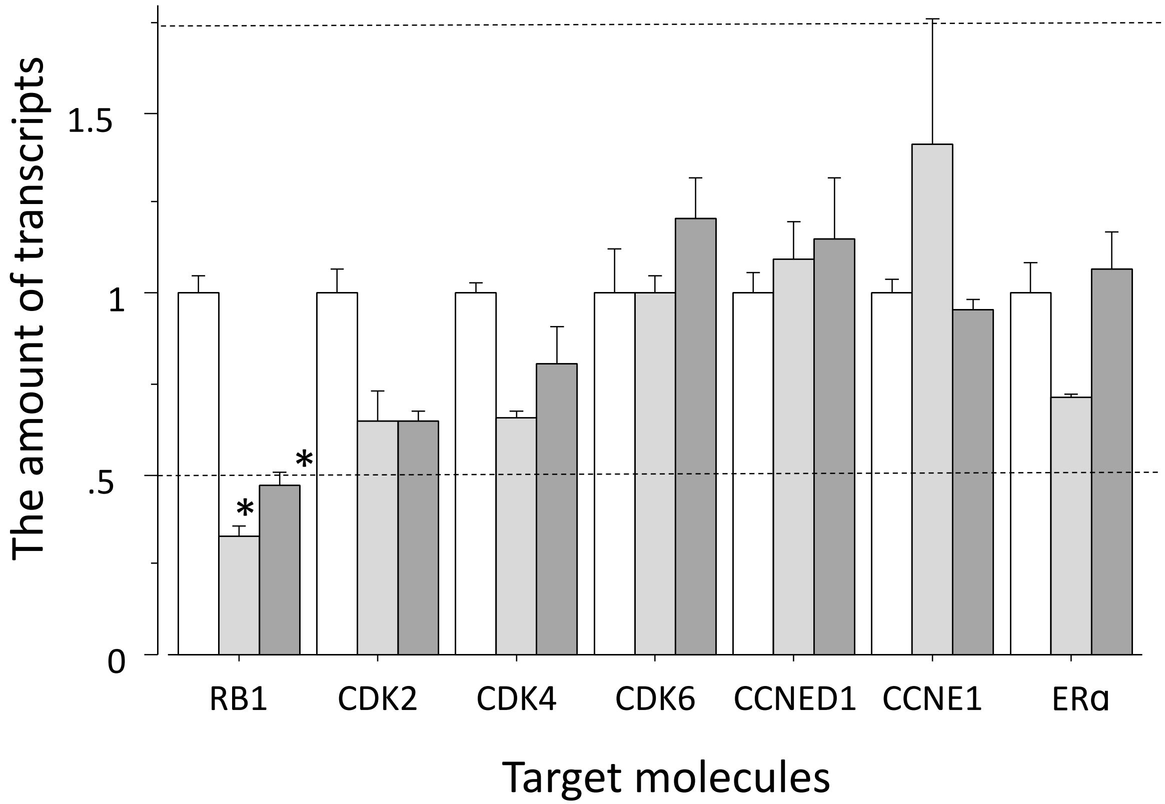

Online Resource 2

Expression levels of cell cycle-related molecules and ER-α in MS cells (white bars), MR-P cells (light grey bars) and MR-A cells (dark grey bars) of the MCF-7 model (a). Those in KS cells (white bars), KR-P cells (light grey bars) and KR-A cells (dark grey bars) of the KPL-1 model (b). Expression levels were measured by RT-PCR as described in Materials and Methods. Values were analyzed after normalization to the controls and expressed as the mean ± SE. The expression level of each molecule in control cells was defined as 1. A change in the transcript amount to more than 2 or less than 0.5 was considered significant (*).
