# Supplementary material for: Resistance to cyclin-dependent kinase (CDK) 4/6 inhibitors confers cross-resistance to other CDK inhibitors but not to chemotherapeutic agents in breast cancer cells
Source: Breast Cancer. 2020 Aug 28;28(1):206–15. doi: 10.1007/s12282-020-01150-8 (PMC7796879; doi:10.1007/s12282-020-01150-8)
Supplement: Supplementary file 3 — Supplementary file3 (PDF 444 kb) [file 12282_2020_1150_MOESM3_ESM.pdf]

Online Resource 3

Resistance to cyclin-dependent kinase (CDK) 4/6 inhibitors confers cross-resistance to other CDK inhibitors but not to chemotherapeutic agents in breast cancer cells, Breast Cancer, Ogata R, et al., Kawasaki medical School, kure@med.Kawasaki-m.ac.jp

a

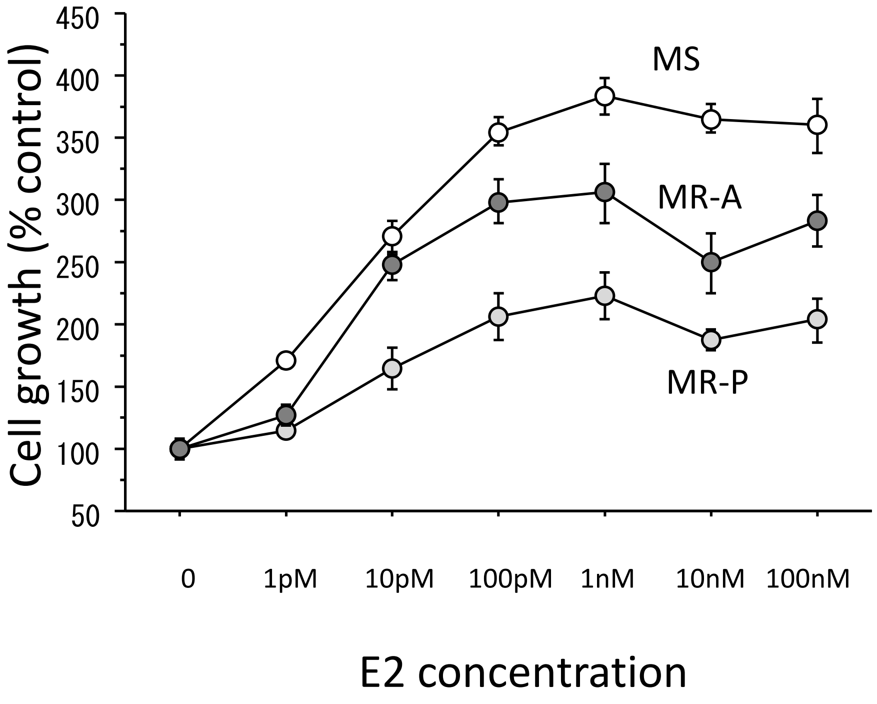

b

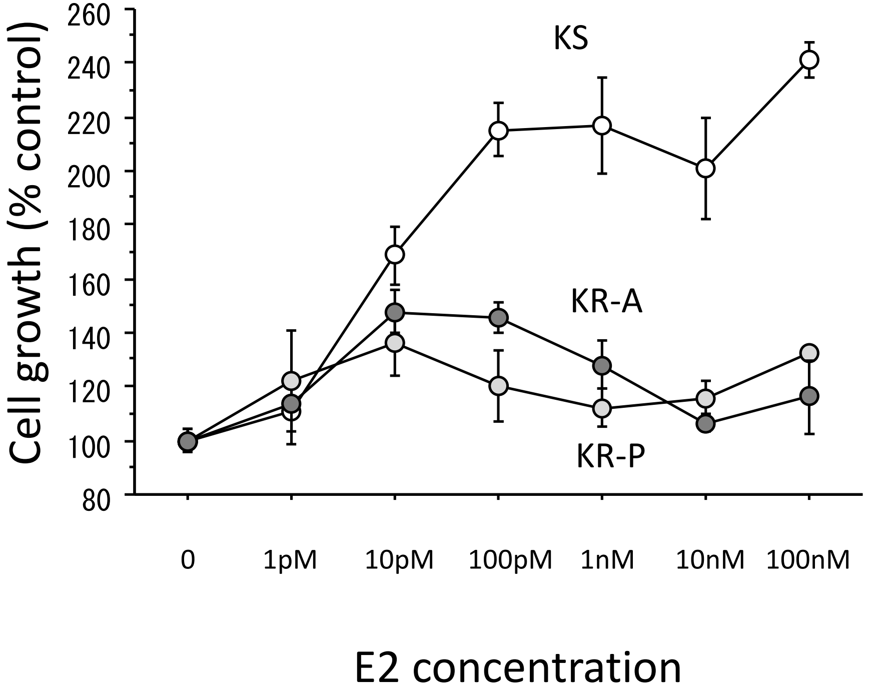

Online Resource 3

Growth-promoting effects of E2 on MS cells (white bars), MR-P cells (light grey bars) and MR-A cells (dark grey bars) in the MCF-7 model (a). Those on KS cells (white bars), KR-P cells (light grey bars) and KR-A cells (dark grey bars) in the KPL-1 model (b). All cells were cultured in the estrogen-deprived medium plus the indicated concentrations of E2 for three days. The cell numbers were measured using the Coulter counter. The values are expressed as the mean ± SE of the % control.
